# Microplastics enhance the risk of cross-genus dissemination of carbapenemase resistance plasmids in ICU patients

**DOI:** 10.3389/fcimb.2026.1781149

**Published:** 2026-03-20

**Authors:** Yongliang Ni, Jianchao Zhang, Cheng Peng, Yong Yang, Yueke Lin, Ziyun Li

**Affiliations:** 1 Department of Urology, Shandong Provincial Third Hospital, Shandong University, Jinan, China; 2 Department of Urology, Shandong Public Health Clinical Center, Shandong University, Jinan, China; 3 Department of Clinical Laboratory, The Second Qilu Hospital of Shandong University, Shandong University, Jinan, China; 4 Shandong Provincial Maternal and Child Health Care Hospital Affiliated to Qingdao University, Jinan, China; 5 School of Public Health, Shandong University, Jinan, China

**Keywords:** antibiotic resistance, carbapenem-resistant enterobacteriaceae, clinical microbiology, intensive care unit, microplastics

## Abstract

**Background:**

The emergence of carbapenem-resistant Enterobacterales (CRE) in intensive care units (ICUs) poses a critical global health threat. Environmental factors within hospitals, including microplastic (MP) pollution derived from degraded medical plastics, are potential yet underexplored contributors to the dissemination of antibiotic resistance. This study aimed to investigate whether MPs can accelerate the horizontal transfer of clinically relevant carbapenemase plasmids among CRE pathogens prevalent in ICUs.

**Methods:**

Representative CRE isolates and epidemic carbapenemase-producing plasmids were co-incubated with environmentally relevant concentrations of characterized MPs. Conjugation frequencies were quantified under simulated ICU conditions, including standard and hyperglycemic media. The influence of MPs on recipient biofilm formation—a key facilitator for genetic exchange—was assessed using crystal violet assays and confocal microscopy. Plastic-free conditions were set as controls.

**Results:**

MPs significantly enhanced the conjugation rates of carbapenemase plasmids between CRE strains (*p* < 0.001). Importantly, the elevated conjugation efficiencies were correlated with potent MP-induced stimulation of biofilm formation in recipient bacteria. Additionally, MPs synergized with the simulated diabetic ICU urine environment, increasing plasmid transfer efficiency by more than 3.96-fold. MPs acted as abiotic surfaces that promoted bacterial aggregation and plasmid exchange.

**Conclusion:**

Our findings reveal that medical plastic-derived MPs serve as novel environmental catalysts for the rapid dissemination of carbapenem resistance within ICUs. By significantly enhancing biofilm-associated plasmid conjugation—especially in the context of patient comorbidity (hyperglycemia)—MPs constitute an emerging environmental driver that exacerbates the spread of untreatable CRE infections, highlighting the need for urgent mitigation strategies.

## 1 Introduction

The global antimicrobial resistance (AMR) crisis is intensifying, with intensive care units (ICUs) serving as critical hotspots for multidrug-resistant (MDR) pathogens (Brinkac et al., 2017; Charalampous et al., 2021; Naddaf, 2024; Stronger commitment and faster action against antimicrobial resistance, 2024). ICU patients account for 32.6% of hospital-acquired resistant infections and face a 4.2-fold higher infection-related mortality than general ward patients (Antimicrobial Resistance Collaborators, 2022). Carbapenem-resistant Enterobacterales (CRE), designated by the WHO as critical-priority pathogens, present severe clinical challenges when exhibiting difficult-to-treat resistance (DTR), defined as non-susceptibility to all first-line bactericidal agents (Kadri et al., 2018; WHO updates list of drug-resistant bacteria most threatening to human health, ). While antibiotic development continues, the rapid emergence of overlapping resistance mechanisms—evidenced by *in vitro* studies showing ESKAPE (*Enterococcus faecium*, *Staphylococcus aureus*, *Klebsiella pneumoniae*, *Acinetobacter baumannii*, *Pseudomonas aeruginosa*, and *Enterobacter* spp.) pathogens developing pan-resistance to clinical and developmental antibiotics within 60 days (Daruka et al., 2025)—threatens therapeutic efficacy.

Environmental co-factors are increasingly recognized as drivers of AMR transmission. Microplastics (MPs), defined as plastic particles <5 mm in size, permeate global ecosystems (Wang et al., 2021; Stapleton et al., 2023; Stoett et al., 2024) and enter humans via ingestion, inhalation, or medical procedures (Florance et al., 2022; Wu et al., 2022; Wolff et al., 2023; Saha and Saha, 2024; Wang et al., 2024; Winiarska et al., 2024; Li et al., 2025d; Nihart et al., 2025). Critically, intravenous administration introduces MPs directly into the circulation, with kidney accumulation enabling urinary excretion—particularly concerning for catheterized ICU patients (Leslie et al., 2022; Pironti et al., 2022; Liu et al., 2024). According to existing research, MPs with particle sizes ranging from 4 to 15 μm are commonly detected in urine samples from healthy adults (Pironti et al., 2022). Among MPs with diameters ≥300 nm, the concentration range is approximately 0.1 to 10 μg/mL (Ji et al., 2025). Notably, exposure levels may be significantly elevated in specific populations, such as neonates in intensive care units, due to the use of medical devices. It has been reported that the average exposure dose of MPs in these infants could be 10 to even 1000 times higher than that in healthy children (Bernard et al., 2021). Concentrations of microplastic particles exceeding 700 nm in the blood of healthy individuals measure approximately 1.6 μg/L (Leslie et al., 2022), and their transmission into the bloodstream via infusion systems has been confirmed (Li et al., 2025a). Huang et al.’s recent study revealed that commercially available infusion bags release 1-62 μm MPs (predominantly 1-10 μm) even after filtration measures (Huang et al., 2025). Thus, intravenous infusion may typically introduce tens of thousands of microplastic particles directly into human blood. Consequently, ICU patients requiring frequent infusions may exhibit higher microplastic concentrations in their blood (Casella et al., 2025; Li et al., 2025a; Adatia, 2026). MP toxicity mechanisms, including oxidative stress and inflammation (Huang et al., 2024), may synergize with co-contaminants like antibiotics to exacerbate AMR (Bank et al., 2020; G. et al., 2025).

Specific size-dependent effects of microplastics on the conjugation and transfer of mobile antibiotic resistance genes (mARGs) in multidrug-resistant clinical bacteria under simulated clinical conditions (e.g., within biofilms, coexisting with disinfectants) remain poorly understood. Medical microplastics, the main source of microplastic contamination in clinical settings, exhibit a wide range of particle sizes, but their regulatory effects on ARG transfer in biofilm microenvironments have not been systematically clarified (Canellas et al., 2023; Khan et al., 2025). Furthermore, the role of medical plastic-derived MPs in facilitating the horizontal transfer of carbapenemase resistance plasmids among clinically relevant pathogens in ICUs remains underexplored. Horizontal gene transfer (HGT) via conjugation is a major mechanism for AMR dissemination, allowing resistance genes to spread across bacterial species and genera (Li et al., 2021, 2025c; Ding et al., 2024; Hernandez-Beltran et al., 2024; Djermoun et al., 2025). Biofilm formation is a key facilitator of conjugation, as bacterial aggregation enhances cell-to-cell contact and genetic exchange (Djermoun et al., 2025). Our previous study have shown that low-dose MPs can regulate biofilm formation and alter the conjugation frequency of laboratory-engineered strains (*Escherichia coli*) (Li et al., 2025d). However, its effects on clinical highly drug-resistant strains remain unclear. Given the high prevalence of medical plastics in ICUs (e.g., catheters, intravenous lines) and the persistent MP pollution they generate, we hypothesized that MPs act as abiotic surfaces to promote biofilm formation and accelerate the conjugation of carbapenemase resistance plasmids among CRE strains. Additionally, we investigated the synergistic effect of hyperglycemic conditions (simulating diabetic comorbidity, a common ICU patient characteristic) on MP-mediated AMR dissemination. This study provides critical insights into an overlooked environmental driver of CRE spread and informs targeted infection control strategies for the post-antibiotic era.

## 2 Materials and methods

### 2.1 Sample collection and bacterial isolation

**Figure 1:**
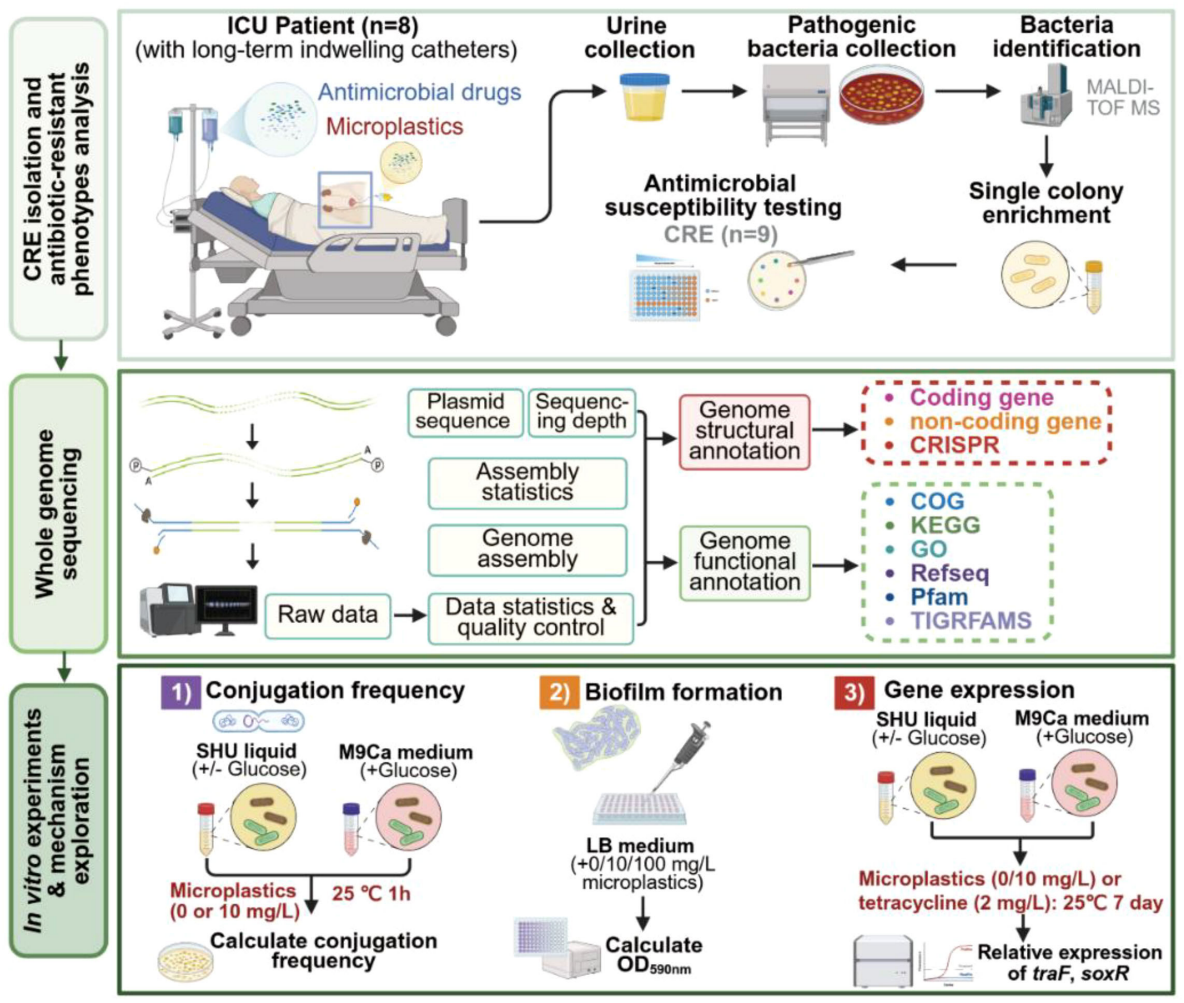
Schematic diagram of the experimental analysis process.

This study was approved by the Ethics Committee of Shandong Provincial Third Hospital (approval number: KYLL-2023096). All procedures involving human samples were conducted in compliance with relevant ethical guidelines and regulations. Urine samples were collected from eight ICU patients with long-term indwelling catheters (≥7 days) during routine laboratory testing (**Figure 1**). Bacterial isolation was performed using standard culture methods, and CRE strains were identified via MALDI-TOF MS (matrix-assisted laser desorption/ionization time-of-flight mass spectrometry) and antimicrobial susceptibility testing.

### 2.2 Strains, culture medium, and reagents

The experimental strains used in this study included nine clinically isolated CRE, a chloramphenicol-resistant *Escherichia coli* strain PBCKS preserved in the laboratory, and two standard strains, *E. coli* ATCC 25922 and *Staphylococcus aureus* ATCC 25923. All strains were stored in LB liquid medium supplemented with 20% glycerol and cryopreserved at -80°C in an ultra-low temperature refrigerator; prior to the experiment, they were revived and activated on LB agar plates to ensure the purity and viability of the strains.

The culture media used in this study were either commercially available products or self-prepared according to standard formulas: Among them, LB liquid medium, LB agar plates, and mannitol agar medium were all purchased from Qingdao Haibo Biotechnology Co., Ltd. (Qingdao, China); the standard media for the normal culture condition group included sterile artificial urine SHU medium (Artificial Urine SHU, Sterile, Solarbio, pH=5.4) and M9Ca medium (Sangon Biotech (Shanghai) Co., Ltd.). Hyperglycemic media were prepared by adding glucose to SHU medium and M9Ca medium respectively to a final concentration of 20% (w/v), which was used to simulate the hyperglycemic microenvironment of urine from patients in the ICU with diabetes mellitus. All culture media underwent sterility verification (incubated at 37°C for 24 h with no contaminating bacterial growth) before use to avoid interference of contamination on the experimental results.

The main reagents used in this study were of analytical grade or bioreagent grade, among which all antibiotics were purchased from Shanghai Yuanye Biotechnology Co., Ltd. (Shanghai, China). All antibiotics were prepared as stock solutions in accordance with the latest standards of the CLSI (Schuetz et al., 2025), stored at -20°C in the dark, and diluted to the required working concentration before use.

### 2.3 Antimicrobial susceptibility testing

As described previously (Hu et al., 2023), antimicrobial susceptibility was determined using the broth microdilution method and Kirby Bauer disk diffusion method against a panel of antibiotics, including penicillins, cephalosporins, carbapenems, aminoglycosides, quinolones, and tetracyclines. Strains were classified as resistant (R) or sensitive (S) based on CLSI guidelines (Schuetz et al., 2025).

### 2.4 Genome sequencing and annotation

Genomic DNA was extracted from pure cultures of nine CRE isolates using a commercial DNA extraction kit (Qiagen, catalog no. 51904). Nanopore long-read sequencing was performed with the PromethION sequencer (Oxford Nanopore Technologies) (Branton et al., 2008). Based on sequencing-by-synthesis (SBS) technology, DNA libraries were sequenced on the Illumina HiSeq high-throughput sequencing platform to generate raw data. After quality control of the raw sequencing data, *de novo* assembly of the bacterial genomes was conducted using Unicycler (v0.4.9) (Wick et al., 2017) and Pilon (v1.23) (Bankevich et al., 2012). Gene prediction of the bacteria was performed using Prodigal-2.6.2 (https://github.com/hyattpd/prodigal/wiki), and genome annotation was carried out with Prokka. Functional annotation was implemented against the COG (Tatusov et al., 2000), GO (Ashburner et al., 2000), Pfam (Mistry et al., 2021), and KEGG (Kanehisa et al., 2004) databases. CRISPR arrays, prophages, genomic islands, and secondary metabolite information were identified using MinCED (v0.4.2), PhiSpy (Tatusov et al., 2000) (https://github.com/linsalrob/PhiSpy), IslandViewer 4 (http://www.pathogenomics.sfu.ca/islandviewer/), and antiSMASH, respectively. Plasmid annotation was performed using Prokka, and BLAST (Altschul et al., 1997) was employed to analyze and screen for carbapenemase-encoding genes (e.g., *bla*_KPC_, *bla*_NDM_) and conjugative elements. Genomic circular maps were generated using the R package circlize (Gu et al., 2014).

### 2.5 Microplastic preparation

Polystyrene MPs (PS-MPs) with a diameter of 4 μm were purchased from Shanghai Fengtai Suhua. MPs were sterilized by autoclaving and suspended in sterile water to prepare a stock solution of 10 mg/mL. Working concentrations of 0 μg/mL (control), 10 μg/mL, and 100 μg/mL were prepared by diluting the stock solution in culture medium.

### 2.6 Conjugation assays

Conjugation assays were performed using donor CRE strains (harboring carbapenemase plasmids) and recipient strains (*Escherichia coli* PBCKS or *Staphylococcus aureus ATCC25923*). As described previously (Li et al., 2021, 2025c), donor and recipient strains were cultured to mid-log phase (OD600 = 0.5-0.6) in LB broth. Equal volumes of donor and recipient cultures (1 mL each) were mixed, and MPs were added to the mixture at final concentrations of 0, 10 μg/mL, or 100 μg/mL. The mixture was incubated at 37 °C for 4 h without shaking. Conjugants were selected on LB agar plates supplemented with appropriate antibiotics (e.g., carbapenems for donor selection, rifampicin for recipient selection). Conjugation frequency was calculated as the number of conjugants per recipient colony-forming unit (CFU). Assays were performed in triplicate under two culture conditions: standard medium (SHU and M9Ca) and hyperglycemic medium (SHU + 20% glucose, M9Ca + 20% glucose) to simulate diabetic ICU urine.

### 2.7 Biofilm formation assays

Biofilm formation was assessed using the crystal violet staining method as described previously (Li et al., 2025d). Bacterial cultures (100 μL) were inoculated into 96-well plates containing culture medium with or without MPs (0 μg/mL or 10 μg/mL) and incubated at 37 °C for 24 h. After removing planktonic cells, wells were washed three times with PBS, fixed with methanol for 15 min, and stained with 0.1% crystal violet for 30 min. Excess stain was washed away, and the plate was air-dried. Bound crystal violet was solubilized with 33% acetic acid, and absorbance was measured at 590 nm (OD590) using a microplate reader.

### 2.8 Gene expression analysis

Quantitative real-time PCR (qRT-PCR) was used to analyze the expression of *soxR* (oxidative stress-related gene) and *traF* (conjugation-related gene) (**Table 1**). Bacterial strains were cultured in medium with or without MPs (10 μg/mL) or tetracycline (2 μg/mL, positive control) at 25 °C for 7 days. Total RNA was extracted using TRIzol reagent, and cDNA was synthesized using a reverse transcription kit (Vazyme, China). qRT-PCR was performed using SYBR Green Master Mix (Vazyme, China) on a StepOnePlus Real-Time PCR System. The *16SrRNA* gene was used as an internal reference, and relative gene expression was calculated using the 2^-ΔΔCt^ method.

**Table 1:** Primer sequences used in this study.

Gene	Primer	Sequence (5’ to 3’)	Tm (°C)
*bla*_NDM1_	F	GCCCAGATCCTCAACTGGAT	60
	R	CGCATTGGCATAAGTCGCAA	
*bla*_KPC_	F	TTACGGCAAAAATGCGCTGG	60
	R	TCCAGACGGAACGTGGTATC	
*soxR*	F	GGCGACCATTGGTGAAGCGT	60
	R	CAATCACTGCGCGAAAGGCA	
*traF*	F	GGCAACCTCGTCGCCTTTA	60
	R	GCAAGTCGGCGTGTTTTCG	
*16SrRNA*	F	CCCAGATGGGATTAGCTTGT	60
	R	TCTGGACCGTGTCTCAGTTC	

### 2.9 Statistical analysis

All statistical analyses were performed using R software. Data are presented as mean ± standard deviation (SD) of three independent experiments. Differences between groups were analyzed using two-tailed Student’s t-test. *P*-values <0.05 were considered statistically significant (*P < 0.05, **P < 0.01, ***P < 0.001).

## 3 Results

Nine CRE strains, including carbapenem-resistant *Klebsiella pneumoniae* (CRKP) and carbapenem-resistant *E. coli* (CREC), were isolated from eight ICU patients with long-term indwelling catheters (**Figure 1**). Antimicrobial susceptibility testing results showed that all strains were resistant to multiple antimicrobials, including carbapenems (e.g., imipenem, meropenem), cephalosporins, and aminoglycosides. In contrast, some strains exhibited susceptibility to tetracyclines (e.g., doxycycline) (**Table 2**; **Supplementary Table 1**). Additionally, the isolated strains exhibited high antimicrobial susceptibility to last-resort antimicrobials such as tigecycline and colistin (**Table 2**).

**Table 2:** ICU patient sources and antimicrobial susceptibility profiles of nine CRE strains.

Strain	Diseases-gender-age (year)	ESBL	Antimicrobial susceptibility	
			Resistance	Sensitive
CREC-U1	Severe pneumonia-Female-53	Neg	AMP, CFZ, GM, CAZ/AVI, AXL, AMP/SULB, CEFU, FEP, AMX/CLAV,PIP/TAZ, CAZ,CDX,CRO,CSULB, TOB, ETP,IMP,TIC/CLAV,MER, CIP,AMK,SMT/TMP,LVX	DOX, MIN, COL, TIG
CREC-U3	Pneumonia- Male-52	Neg	AMP, CFZ, GM, AXL, CDX, AMP/SULB, AMX/CLAV, PIP/TAZ, CEFU, CAZ, CRO, CSULB,FEP, ETP, IMP, TIC/CLAV, MER, TOB, CIP, DOX, SMT/TMP, LVX	CAZ/AVI,MIN, COL, AMK, TIG
CRKP-1	Tetraplegia- Male-15	Neg	AMP, CFZ, GM, AXL, CEFU, AMP/SULB, PIP/TAZ, CAZ, CDX, CRO, CSULB, FEP, ETP, IMP, TIC/CLAV, MER, TOB, CIP, DOX, MIN, AMK, SMT/TMP, LVX	CAZ/AVI,COL, TIG
CREC-2	Heart failure- Male-67	Neg	AMP, CFZ, GM, CAZ/AVI, AXL, AMP/SULB, CEFU, AMX/CLAV,PIP/TAZ, CAZ, CDX, CRO, CSULB, FEP, ETP, IMP, TIC/CLAV, MER, TOB, CIP, DOX, AMK, LVX, SMT/TMP	MIN, COL, TIG
CRKP-3	Tetraplegia-Female-63	Neg	AMP, CFZ, GM, AXL, AMP/SULB, AMX/CLAV, PIP/TAZ, CEFU, CAZ, CRO, CSULB, ETP, IMP, TIC/CLAV, MER	CAZ/AVI,CDX,TOB,CIP, DOX, MIN, COL, AMK, TIG, SMT/TMP, LVX
CREC-32	Tetraplegia-Female-39	Neg	AMP, CFZ, GM, CAZ/AVI, AXL, AMP/SULB, CEFU, AMX/CLAV, PIP/TAZ, CAZ, CDX, CRO,CSULB,FEP, ETP, IMP, TIC/CLAV, MER, TOB, CIP, DOX, AMK, SMT/TMP, LVX	MIN,COL,TIG
CREC-36	Severe pneumonia-Female-53	Neg	AMP, CFZ, GM, CAZ/AVI, AXL, AMP/SULB, CEFU, AMX/CLAV, PIP/TAZ, CAZ, CDX, CRO, CSULB, FEP, ETP, IMP, TIC/CLAV, MER, TOB, CIP, AMK, SMT/TMP, LVX	DOX,MIN, COL, TIG
CRKP-58	Dysphagia- Male-68	Pos	AMP, CFZ, AXL, AMP/SULB, AMX/CLAV, PIP/TAZ, CEFU, CRO, ETP, IMP, TIC/CLAV, MER	GM,CAZ/AVI,CAZ,CDX,TOB,CIP, DOX, MIN, COL, AMK, TIG, SMT/TMP, LVX
CRKP-59	Paraplegia-Female-65	Neg	AMP, CFZ, GM, AXL, CEFU, AMP/SULB, AMX/CLAV, PIP/TAZ, CAZ, CDX, CRO, CSULB, FEP, ETP, IMP, TIC/CLAV, MER, TOB, CIP, DOX, AMK, LVX	CAZ/AVI,MIN, COL, TIG, SMT/TMP

Neg, Negative; Pos, Positive; AMP, Ampicillin; CFZ, Cefazolin; GM, Gentamicin; AXL, Aztreonam; CAZ/AVI, Ceftazidime/Avibactam; AMX/CLAV, Amoxicillin/Clavulanic acid; CDX, Cefoxitin; TOB, Tobramycin; CEFU, Cefuroxime; DOX, Doxycycline; PIP/TAZ, Piperacillin/Tazobactam; CIP, Ciprofloxacin; LVX, Levofloxacin; SMZ/TMP, Trimethoprim/Sulfamethoxazole; CRO, Ceftriaxone; ETP, Ertapenem; TIC/CLAV, Ticarcillin/Clavulanic acid; CAZ, Ceftazidime; CSULB, Cefoperazone/Sulbactam; FEP, Cefepime; IMP, Imipenem; MER, Meropenem; AMK, Amikacin; MIN, Minocycline; COL, Polymyxin; TIG, Tigecycline.

**Figure 2:**
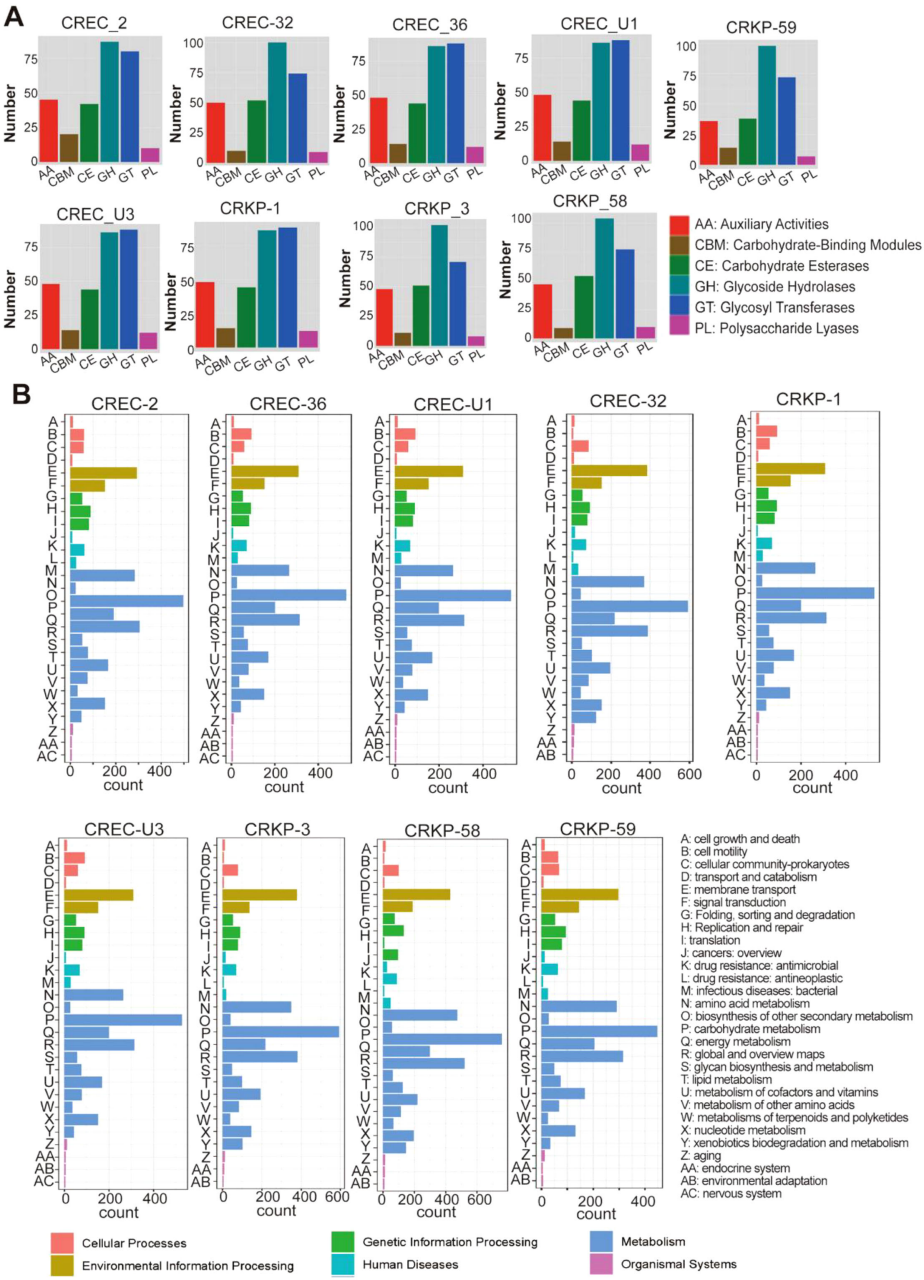
CAZy and KEGG-Based Functional Profiling of Nine CRE Strains. **(A)** CAZy Annotation of Enzyme Families in nine CRE; **(B)** Functional classification of gene products in nine CRE strains based on KEGG annotations.

Complete genome sequences of nine CRE strains were obtained via Nanopore sequencing and *de novo* assembly (**Supplementary Figure S1-S9**; **Supplementary Table 2**). Functional annotation revealed that genes associated with glycoside hydrolases and glycosyltransferases were the most abundant functional categories (**Figure 2A**), accompanied by the enrichment of carbohydrate metabolism pathways (**Figure 2B**)—suggesting that these genes may play roles in biofilm formation and stress adaptation.

**Figure 3:**
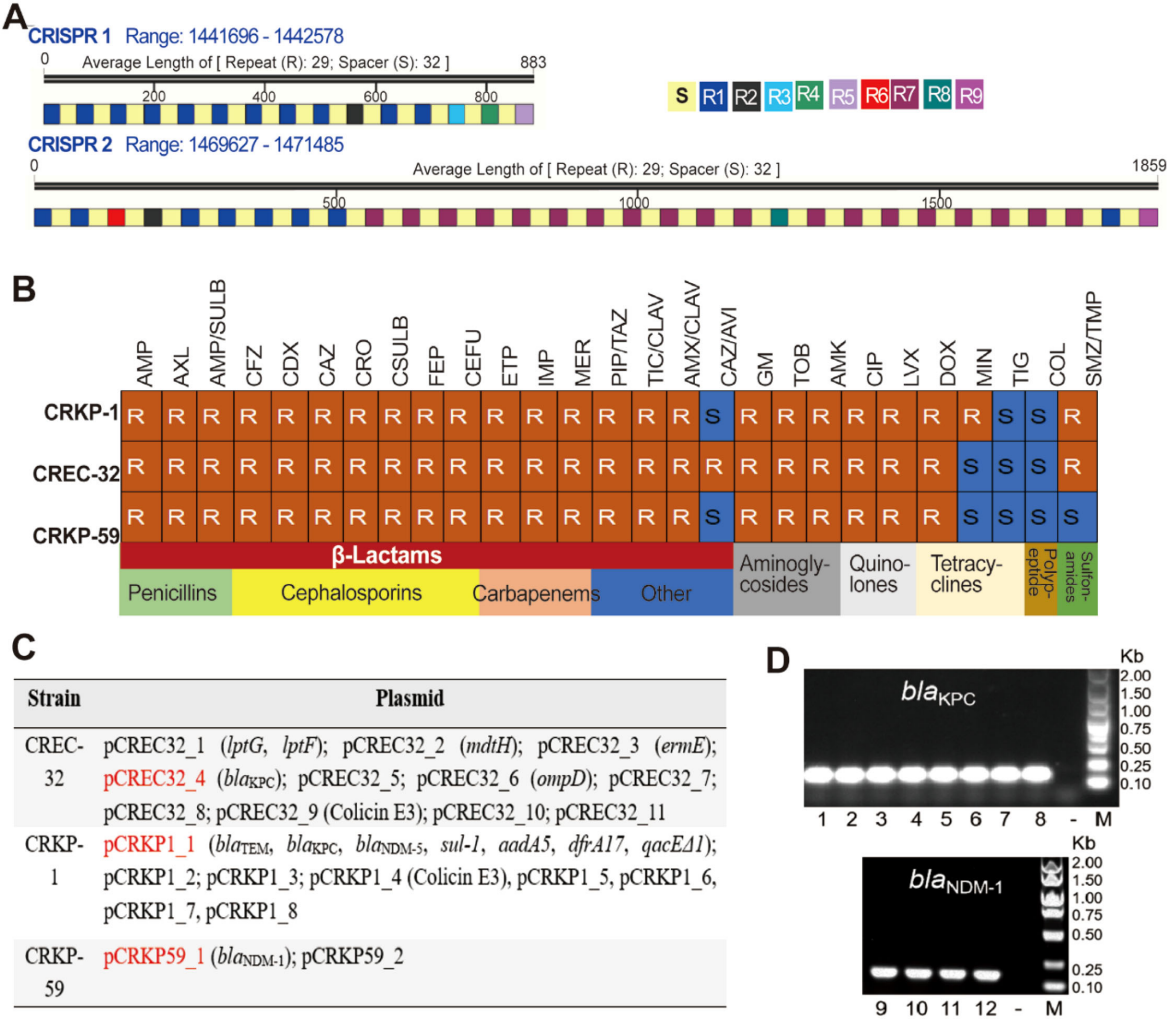
Multifaceted Analysis of CRE Resistance: CRISPR, Antimicrobial Susceptibility, Resistance Plasmids, and Conjugation. **(A)** Phage defense mechanisms in CREC-2 via CRISPR array analysis; **(B)** Antimicrobial susceptibility analysis of CRE with drug resistance plasmids, R: resistance, S: sensitive; **(C)** Key resistance-related genes and potentially conjugative plasmids in CRE drug resistance plasmids (red); **(D)** Conjugation transfer validation of three potential conjugative drug resistance plasmids, Conjugants: 1, 2: *E. coli* (pCREC32_4); 3, 4: *E. coli* (pCRKP1_1); 5, 6: *S. aureus* (pCREC32_4); 7, 8: *S. aureus* (pCRKP1_1); 9, 10: *E. coli* PBCKS (pCRKP59_1); 11, 12: *S. aureus* (pCRKP59_1).

Notably, the CREC-2 strain harbors two CRISPR arrays (**Figure 3A**), suggesting an endogenous defense mechanism against extrinsic genetic elements. Furthermore, prophages and genomic islands were ubiquitous across all strains (**Supplementary Tables S3, S4**), indicating that CRE strains can achieve evolutionary adaptation via gene acquisition and recombination. Specifically, prophages enhance the strains’ environmental survival capacity by mediating the acquisition of novel genes; genomic islands facilitate the strains’ rapid adaptation to stressors (such as antibiotic exposure and nutrient limitation); and the coexistence of the two can induce recombination-driven genetic diversity, further promoting strain evolution.

Among the nine CRE strains, CRKP-1, CRKP-59, and CREC-32 carry antibiotic resistance plasmids, and these three strains exhibited resistance to the vast majority of antibiotics (**Figure 3B**). Further analysis revealed that mARGs (conferring carbapenem resistance) were detected in the resistance plasmids of all three strains; notably, the plasmids carried by CRKP-1 and CREC-32 also contained genes associated with colibactin biosynthesis (**Figure 3C**), which endows them with an enhanced competitive colonization advantage in polymicrobial environments.

Crucially, conjugation assay results demonstrated that plasmid pCRKP59_2 carrying the *bla*_NDM-1_ gene and plasmid pCRKP1_2 carrying the *bla*_NDM-5_ gene could be transferred from *K. pneumoniae* to *S. aureus*; meanwhile, plasmid pCREC32_4 carrying the *bla*_KPC_ gene was able to overcome Gram-type barriers and undergo intergeneric transfer from *E. coli* to *S. aureus* (**Figure 3D**). This indicates that clinical CRE strains possess evolutionary potential to surmount intrinsic host restrictions. Following the interspecies transfer of such plasmids carrying broad-spectrum resistance determinants, their horizontal dissemination in clinical settings can significantly increase the risk of emergence of untreatable MDR pathogens.

**Figure 4:**
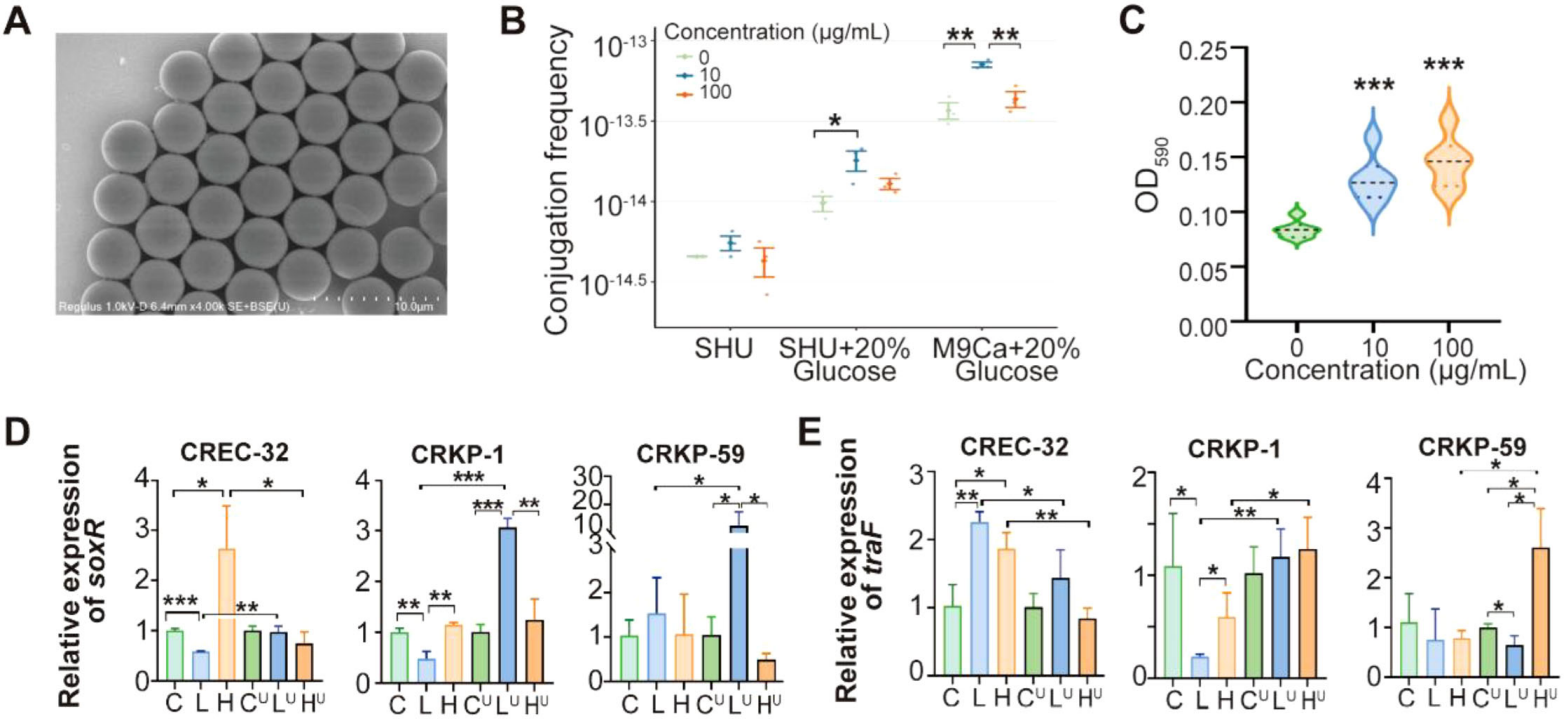
Impact of PS-MPs on Conjugation, Biofilm, and Stress Responses in CREC32 under Varying Nutrients. **(A)** Scanning electron microscopy of polystyrene MPs (PS-MPs); **(B)** Impact of PS-MPs on conjugation transfer of pCREC_4 plasmid under different nutrient conditions; **(C)** MPs-enhanced biofilm formation in CREC32; MPs modulate **(D)** oxidative stress and **(E)** conjugation transfer-related genes in diverse nutrient conditions, C, L and H represent 0, 10 and 100 μg/mL MPs, respectively. The superscript U indicates that the culture medium is SHU supplemented with 20% glucose. *p < 0.05; **p < 0.01; ***p < 0.001.

Subsequently, we further analyzed the conjugation frequencies of conjugative resistance plasmids among three clinical CRE strains under different environmental conditions and stressors. PS-MPs (4 μm, 10 μg/mL) significantly increased the conjugation frequency between CREC32 and *E. coli* PBCKS compared to the plastic-free control (*p* < 0.001) (**Figures 4A, B**). The promotive effect of MPs was amplified in hyperglycemic medium (SHU + 20% glucose), with a 3.96-fold increase in *bla*_KPC_ transfer efficiency (*p* < 0.01) (**Figure 4B**). Higher MP concentrations (100 μg/mL) did not further enhance conjugation frequency, suggesting a concentration-dependent effect. Crystal violet assays showed that MPs (10 μg/mL) robustly stimulated biofilm formation in CREC32, as indicated by a significant increase in OD590 compared to the control (*p* < 0.05) (**Figure 4C**). MPs differentially modulated the expression of *soxR* and *traF* in CREC32 and CRKP strains across culture media. In M9Ca medium, MPs increased gene expression in CREC32 but inhibited it in CRKP at low concentrations. In hyperglycemic SHU medium (20% glucose), MPs significantly promoted the expression of *soxR* and *traF* in CRKP (*p* < 0.05), indicating that hyperglycemic conditions synergize with MPs to enhance oxidative stress and conjugation-related gene expression (**Figures 4D, E**). In summary, MPs in specific environments, particularly under hyperglycemic conditions, can significantly exacerbate plasmid-mediated resistance transmission among clinical drug-resistant strains by upregulating conjugation-related genes and enhancing biofilm formation.

## 4 Discussion

This study demonstrates that MPs derived from the degradation of medical plastics can serve as novel environmental catalysts, promoting the horizontal transfer of carbapenemase resistance plasmids among CRE pathogens in the ICU. In contrast to previous studies focusing primarily on nanoplastic-induced ROS-mediated gene transfer in planktonic bacteria (Li et al., 2025b), the key findings of this work are as follows: micrometer-sized microplastics released from degrading medical devices (e.g., catheters, infusion lines) serve as abiotic surfaces that promote bacterial aggregation and biofilm formation, thereby significantly enhancing plasmid conjugative transfer efficiency. A hyperglycemic environment (simulating diabetic comorbidity) synergizes with microplastics, increasing plasmid transfer efficiency by nearly fourfold. Furthermore, after extended incubation, microplastics may also induce bacterial ROS production, potentially associated with the degradation of microplastics into nanosized particles. These findings expand the understanding of the size-dependent effects of plastic particles (micro- *vs*. nano-) and suggest that the risks posed by microplastics in clinical settings may be underestimated.

The study further highlights its clinical significance: the cross-genus transmission of carbapenemase plasmids—particularly from *Klebsiella* and *E. coli* to *S. aureus*—is of special concern, as it may further limit therapeutic options for ICU-acquired infections. The *bla*_NDM-5_ gene mediates bacterial resistance to the novel antibiotic cefiderocol (not yet available in China) (Simner et al., 2022), and CRE strains isolated from patient urine samples in Chinese hospitals in this study not only carried *bla*_NDM-5_ but also demonstrated that this gene can spread via plasmids across Gram-negative and Gram-positive species, which may accelerate the loss of antibiotic efficacy. Moreover, some CRE strains in this study carried conjugative plasmids encoding both carbapenemases and the colibactin biosynthesis system, enhancing their colonization competitiveness in polymicrobial environments and facilitating the spread of resistance within complex ICU microbial communities.

Mechanistically, MPs enhance conjugation by stimulating biofilm formation, which provides a protected environment for bacterial aggregation and genetic exchange. Gene expression analysis revealed that MPs modulate oxidative stress and conjugation-related genes (e.g., *soxR* and *traF*) in a strain- and environment-dependent manner. Under hyperglycemic conditions, the upregulation of these genes in CRKP was particularly pronounced, suggesting that diabetic patients may face a higher risk of MP-mediated resistance transmission.

Although inline filtration devices are currently used in ICUs, MPs persist in medical equipment and patient blood, underscoring the clinical relevance of these findings. Based on this, we propose the following mitigation strategies: (1) reevaluate medical device materials to reduce microplastic shedding; (2) develop biofilm-penetrating enzymatic cleaners for MPs in ICU settings; (3) establish microplastic biosafety thresholds in critical care environments; and (4) optimize infection control protocols for high-risk patients such as those with diabetes.

Limitations of this study include the use of only one type and size of polystyrene MPs and the exclusive reliance on *in vitro* experiments. Future research should explore the effects of MPs of different materials and sizes and validate these findings through *in vivo* studies. In conclusion, this study provides the direct evidence that medical-derived MPs accelerate the cross-genus spread of carbapenemase resistance plasmids in ICUs, particularly under diabetic comorbidity. These findings position MPs as an emerging environmental driver of the “silent pandemic” of untreatable infections, underscoring the urgent need to integrate this factor into precision infection control frameworks. Addressing microplastic pollution in healthcare settings is crucial for mitigating the AMR crisis and improving patient outcomes in ICUs.

## Data Availability

The datasets presented in this study can be found in online repositories. The names of the repository/repositories and accession number(s) can be found below: https://www.ncbi.nlm.nih.gov/, PRJNA1282339 https://www.ncbi.nlm.nih.gov/, PRJNA1282396.
