# Correction: Microplastics enhance the risk of cross-genus dissemination of carbapenemase resistance plasmids in ICU patients

**DOI:** 10.3389/fcimb.2026.1840192

**Published:** 2026-04-21

**Authors:** Yongliang Ni, Jianchao Zhang, Cheng Peng, Yong Yang, Yueke Lin, Ziyun Li

**Affiliations:** 1 Department of Urology, Shandong Provincial Third Hospital, Shandong University, Jinan, China; 2 Department of Urology, Shandong Public Health Clinical Center, Shandong University, Jinan, China; 3 Department of Clinical Laboratory, The Second Qilu Hospital of Shandong University, Shandong University, Jinan, China; 4 Shandong Provincial Maternal and Child Health Care Hospital Affiliated to Qingdao University, Jinan, China; 5 School of Public Health, Shandong University, Jinan, China

**Keywords:** antibiotic resistance, carbapenem-resistant enterobacteriaceae, clinical microbiology, intensive care unit, microplastics

The affiliations were erroneously given as:

“Yongliang Ni1,2†, Jianchao Zhang1†, Cheng Peng2†, Yong Yang2, Yueke Lin3* and Ziyun Li4,5*

1Department of Urology, Shandong Public Health Clinical Center, Shandong University, Jinan, China, 2Department of Urology, Shandong Provincial Third Hospital, Shandong University, Jinan, China, 3Department of Clinical Laboratory, The Second Qilu Hospital of Shandong University, Shandong University, Jinan, China, 4Shandong Provincial Maternal and Child Health Care Hospital Affiliated to Qingdao University, Jinan, China, 5School of Public Health, Shandong University, Jinan, China”.

The correct affiliations are:

“Yongliang Ni1,2†, Jianchao Zhang1†, Cheng Peng2†, Yong Yang2, Yueke Lin3* and Ziyun Li4,5*

1Department of Urology, Shandong Provincial Third Hospital, Shandong University, Jinan, China, 2Department of Urology, Shandong Public Health Clinical Center, Shandong University, Jinan, China, 3Department of Clinical Laboratory, The Second Qilu Hospital of Shandong University, Shandong University, Jinan, China, 4Shandong Provincial Maternal and Child Health Care Hospital Affiliated to Qingdao University, Jinan, China, 5School of Public Health, Shandong University, Jinan, China”

The original version of this article has been updated.

