# Supplementary material for: Microplastics enhance the risk of cross-genus dissemination of carbapenemase resistance plasmids in ICU patients
Source: Front Cell Infect Microbiol. 2026 Mar 20;16:1781149. doi: 10.3389/fcimb.2026.1781149 (PMC13047061; doi:10.3389/fcimb.2026.1781149)
Supplement: Supplementary file 1 [file DataSheet1.pdf]

Supplementary materials

Microplastics enhance the risk of cross-genus dissemination of carbapenemase resistance plasmids in ICU patients

Yongliang Ni[2,*], Jianchao Zhang[1,*], Cheng Peng[2,*],Yong Yang[2], Yueke Lin[3,#], Ziyun Li[4,5,#]

[1] Department of Urology, Shandong Provincial Third Hospital, Shandong University, Jinan, China

[2] Department of Urology, Shandong Public Health Clincial Center, Shandong University, Jinan, China.

[3] Department of clinical laboratory, The Second Qilu Hospital of Shandong University, Shandong University, Jinan, China.

[4] School of Public Health, Shandong University, Jinan, China

[5] Shandong Provincial Maternal and Child Health Care Hospital Affiliated to Qingdao University, Jinan, China

* Yongliang Ni, Jianchao Zhang and Cheng Peng contributed equally to this work.

[#] Corresponding Authors: Yueke Lin (lin790832736@163.com) and Ziyun Li (liziyun@sdu.edu.cn)

Contents

Figure S1..2

Figure S2..3

Figure S3..4

Figure S4..5

Figure S5..6

Figure S6..7

Figure S7..8

Figure S8..9

Figure S9..10

Table S2..11

Table S4..11

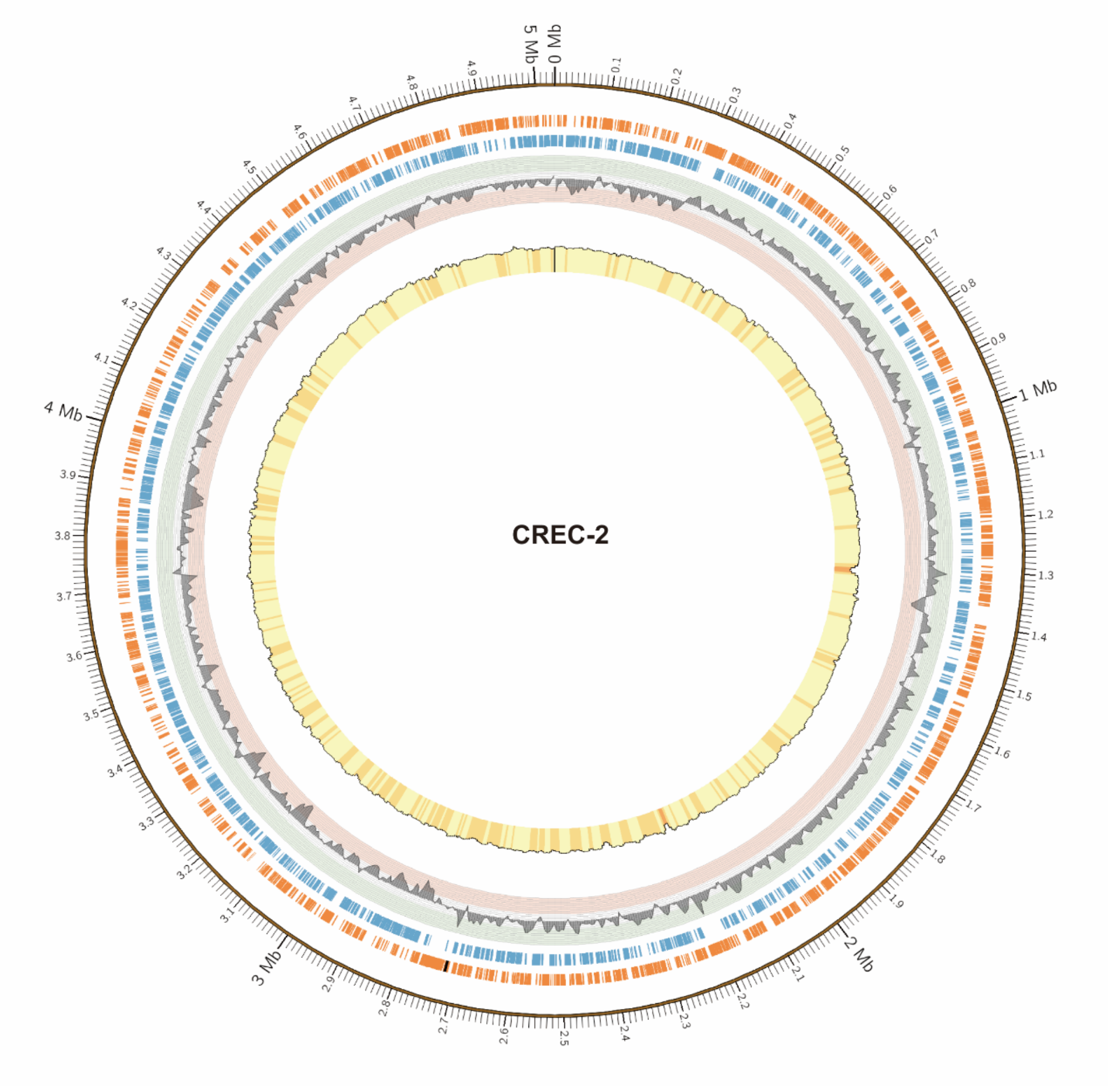

Fig. S1 The circular genome map of CREC-2 is structured as follows, from the outermost to the innermost layer: First circle: Displays the genome sequence information. Second circle: Shows the CDS, rRNA, and tRNA regions of the reference genome, with the outer layer representing the positive strand and the inner layer the negative strand. Third circle: Presents the GC skew curve of the genome sequence, calculated by a sliding window of 2,000 bp to determine the average GC content, along with a dashed line indicating the GC skew baseline of 0. Fourth circle: Depicts the GC content curve of the genome sequence, also calculated by a sliding window of 2,000 bp to determine the average GC content, with a dashed line showing the average GC content of the reference genome.

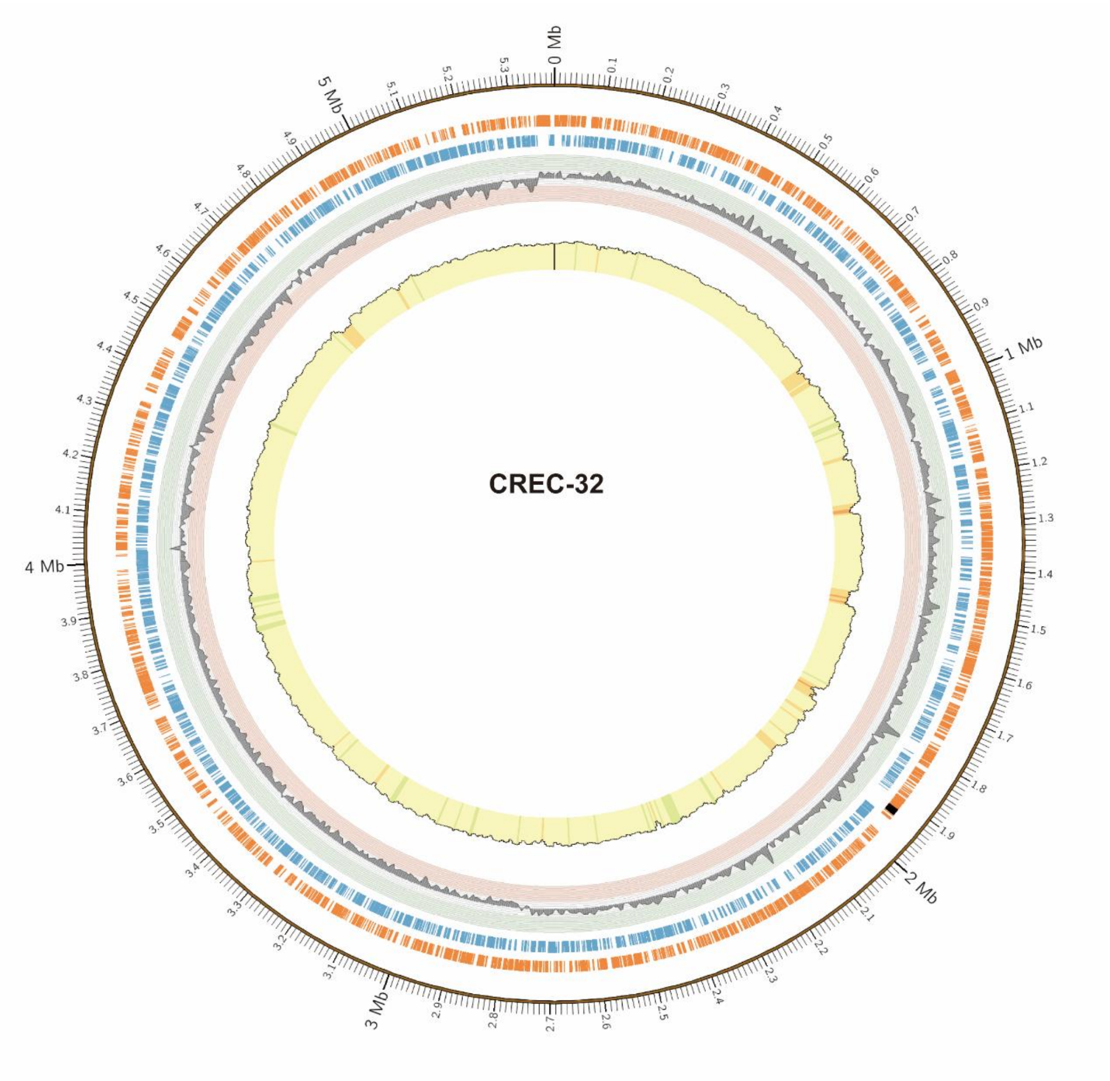

Fig. S2 The circular genome map of CREC-32 is structured as follows, from the outermost to the innermost layer: First circle: Displays the genome sequence information. Second circle: Shows the CDS, rRNA, and tRNA regions of the reference genome, with the outer layer representing the positive strand and the inner layer the negative strand. Third circle: Presents the GC skew curve of the genome sequence, calculated by a sliding window of 2,000 bp to determine the average GC content, along with a dashed line indicating the GC skew baseline of 0. Fourth circle: Depicts the GC content curve of the genome sequence, also calculated by a sliding window of 2,000 bp to determine the average GC content, with a dashed line showing the average GC content of the reference genome.

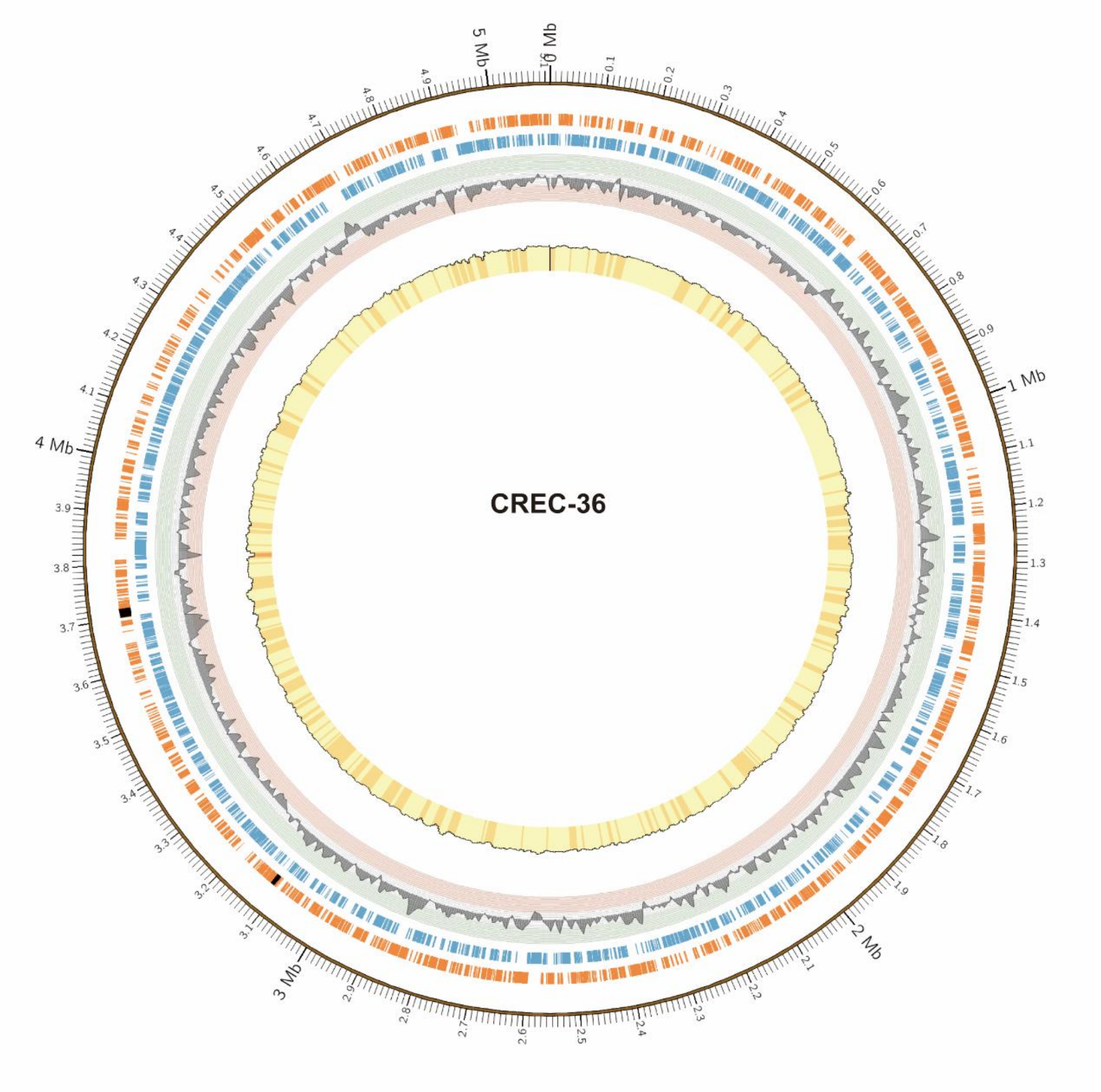

Fig. S3 The circular genome map of CREC-36 is structured as follows, from the outermost to the innermost layer: First circle: Displays the genome sequence information. Second circle: Shows the CDS, rRNA, and tRNA regions of the reference genome, with the outer layer representing the positive strand and the inner layer the negative strand. Third circle: Presents the GC skew curve of the genome sequence, calculated by a sliding window of 2,000 bp to determine the average GC content, along with a dashed line indicating the GC skew baseline of 0. Fourth circle: Depicts the GC content curve of the genome sequence, also calculated by a sliding window of 2,000 bp to determine the average GC content, with a dashed line showing the average GC content of the reference genome.

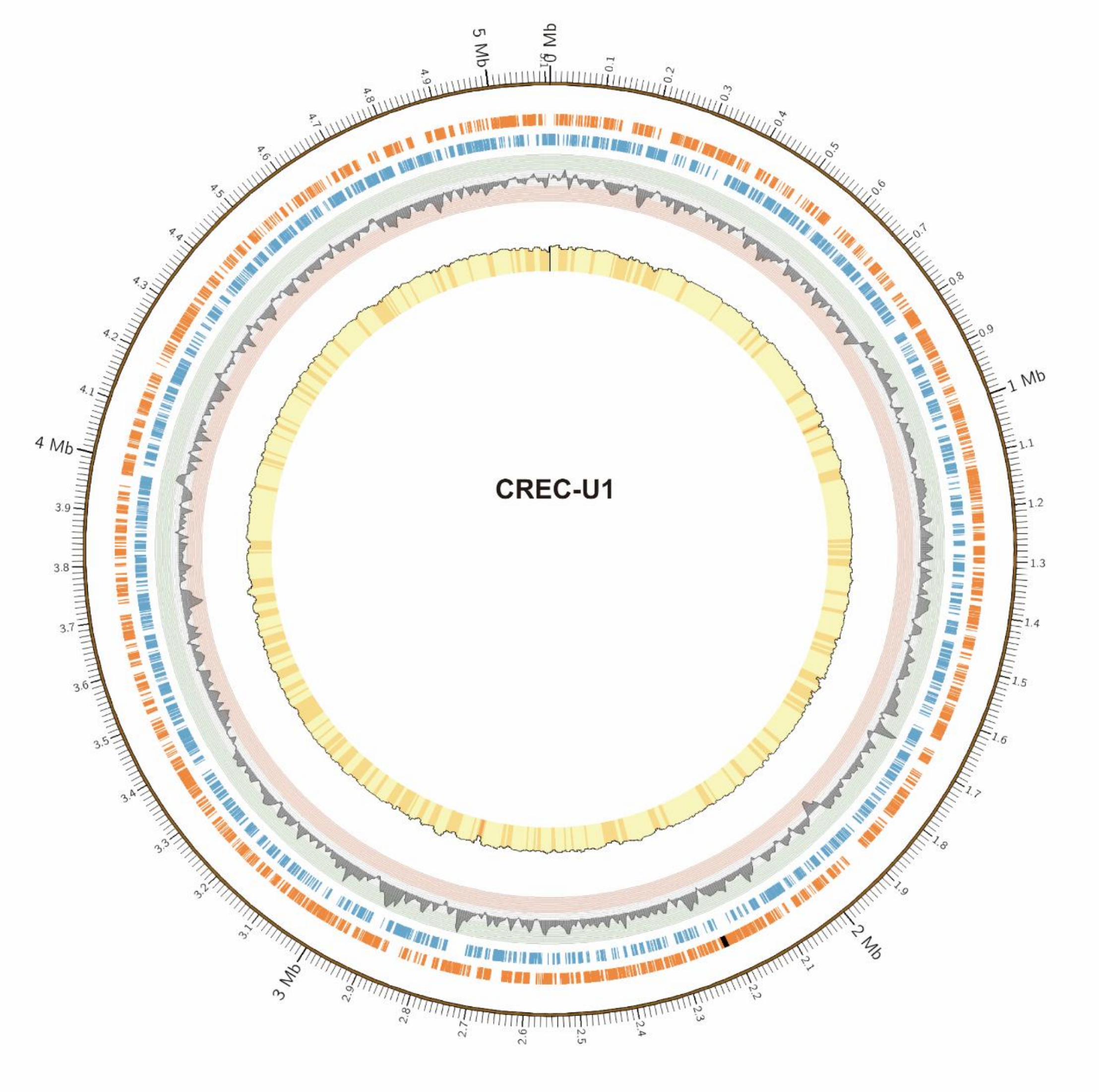

Fig. S4 The circular genome map of CREC-U1 is structured as follows, from the outermost to the innermost layer: First circle: Displays the genome sequence information. Second circle: Shows the CDS, rRNA, and tRNA regions of the reference genome, with the outer layer representing the positive strand and the inner layer the negative strand. Third circle: Presents the GC skew curve of the genome sequence, calculated by a sliding window of 2,000 bp to determine the average GC content, along with a dashed line indicating the GC skew baseline of 0. Fourth circle: Depicts the GC content curve of the genome sequence, also calculated by a sliding window of 2,000 bp to determine the average GC content, with a dashed line showing the average GC content of the reference genome.

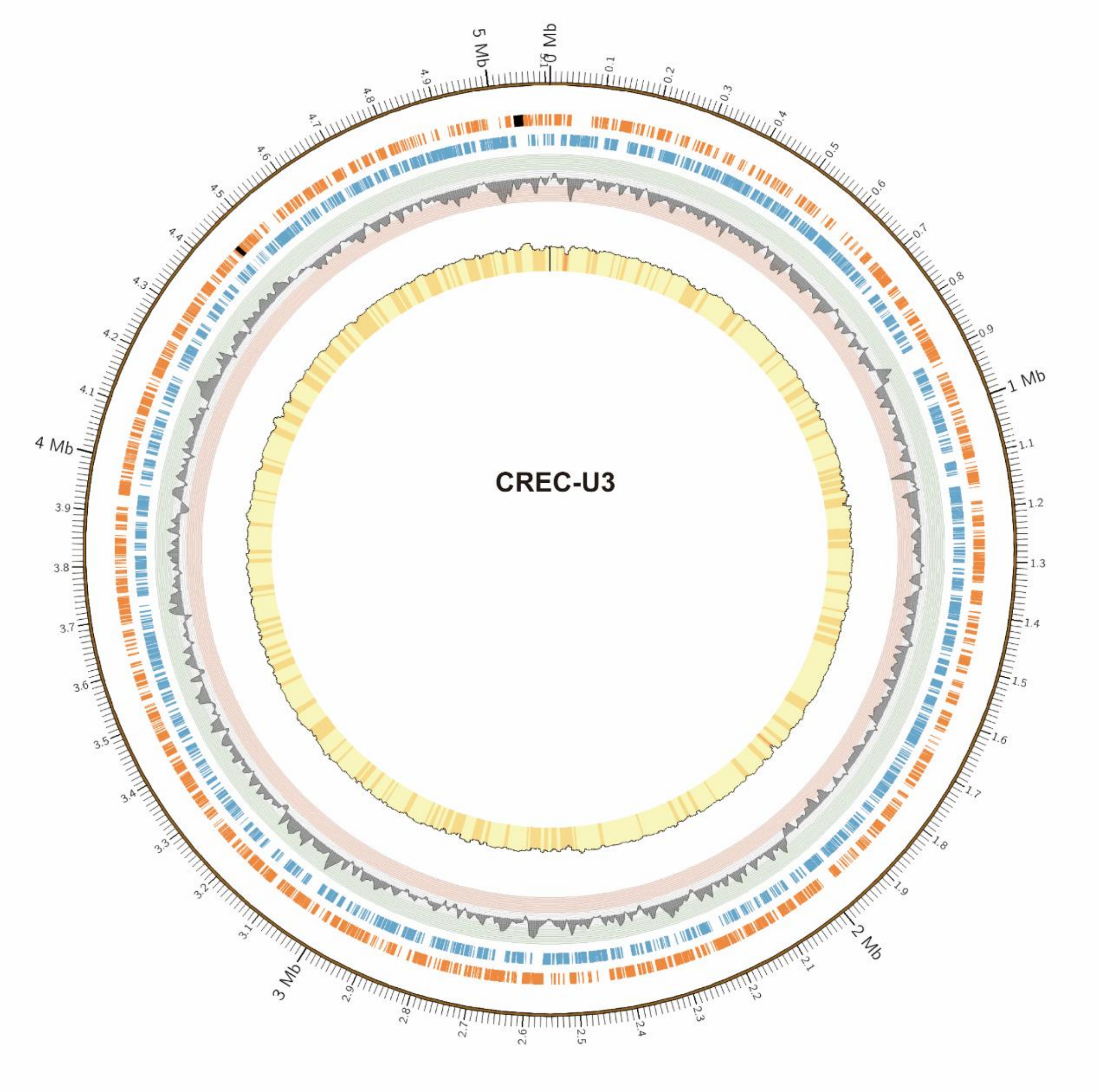

Fig. S5 The circular genome map of CREC-U3 is structured as follows, from the outermost to the innermost layer: First circle: Displays the genome sequence information. Second circle: Shows the CDS, rRNA, and tRNA regions of the reference genome, with the outer layer representing the positive strand and the inner layer the negative strand. Third circle: Presents the GC skew curve of the genome sequence, calculated by a sliding window of 2,000 bp to determine the average GC content, along with a dashed line indicating the GC skew baseline of 0. Fourth circle: Depicts the GC content curve of the genome sequence, also calculated by a sliding window of 2,000 bp to determine the average GC content, with a dashed line showing the average GC content of the reference genome.

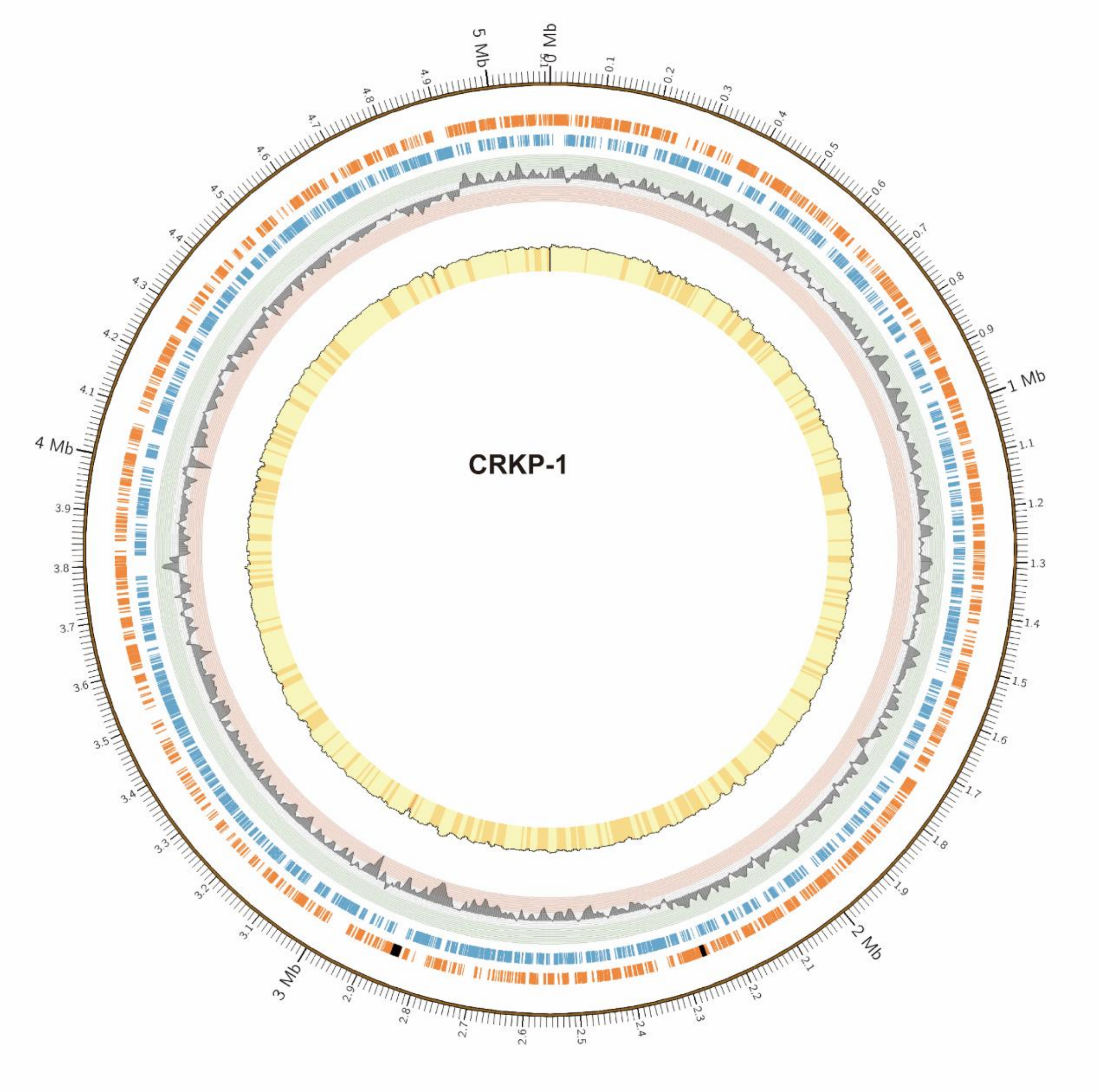

Fig. S6 The circular genome map of CRKP-1 is structured as follows, from the outermost to the innermost layer: First circle: Displays the genome sequence information. Second circle: Shows the CDS, rRNA, and tRNA regions of the reference genome, with the outer layer representing the positive strand and the inner layer the negative strand. Third circle: Presents the GC skew curve of the genome sequence, calculated by a sliding window of 2,000 bp to determine the average GC content, along with a dashed line indicating the GC skew baseline of 0. Fourth circle: Depicts the GC content curve of the genome sequence, also calculated by a sliding window of 2,000 bp to determine the average GC content, with a dashed line showing the average GC content of the reference genome.

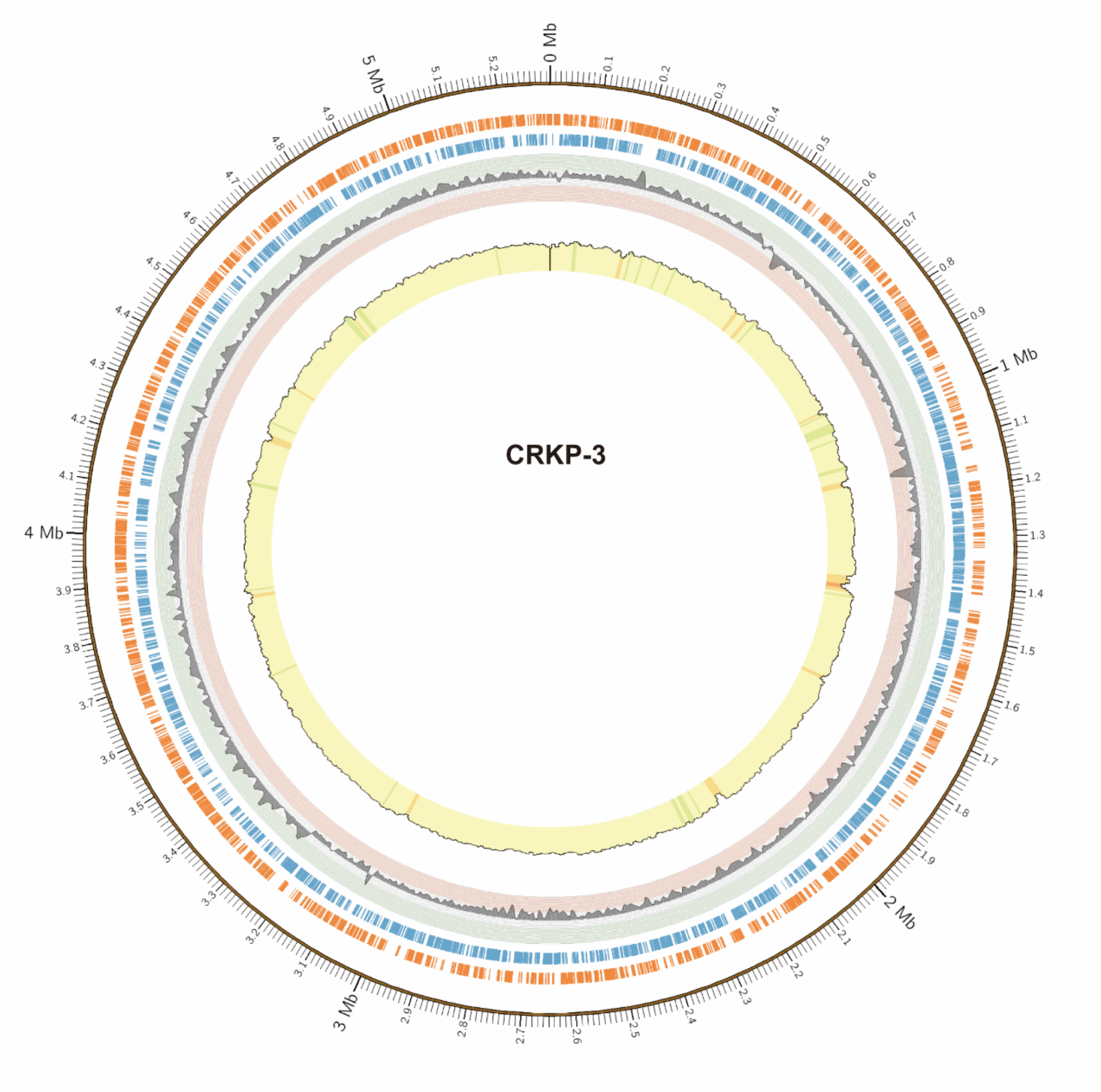

Fig. S7 The circular genome map of CRKP-3 is structured as follows, from the outermost to the innermost layer: First circle: Displays the genome sequence information. Second circle: Shows the CDS, rRNA, and tRNA regions of the reference genome, with the outer layer representing the positive strand and the inner layer the negative strand. Third circle: Presents the GC skew curve of the genome sequence, calculated by a sliding window of 2,000 bp to determine the average GC content, along with a dashed line indicating the GC skew baseline of 0. Fourth circle: Depicts the GC content curve of the genome sequence, also calculated by a sliding window of 2,000 bp to determine the average GC content, with a dashed line showing the average GC content of the reference genome.

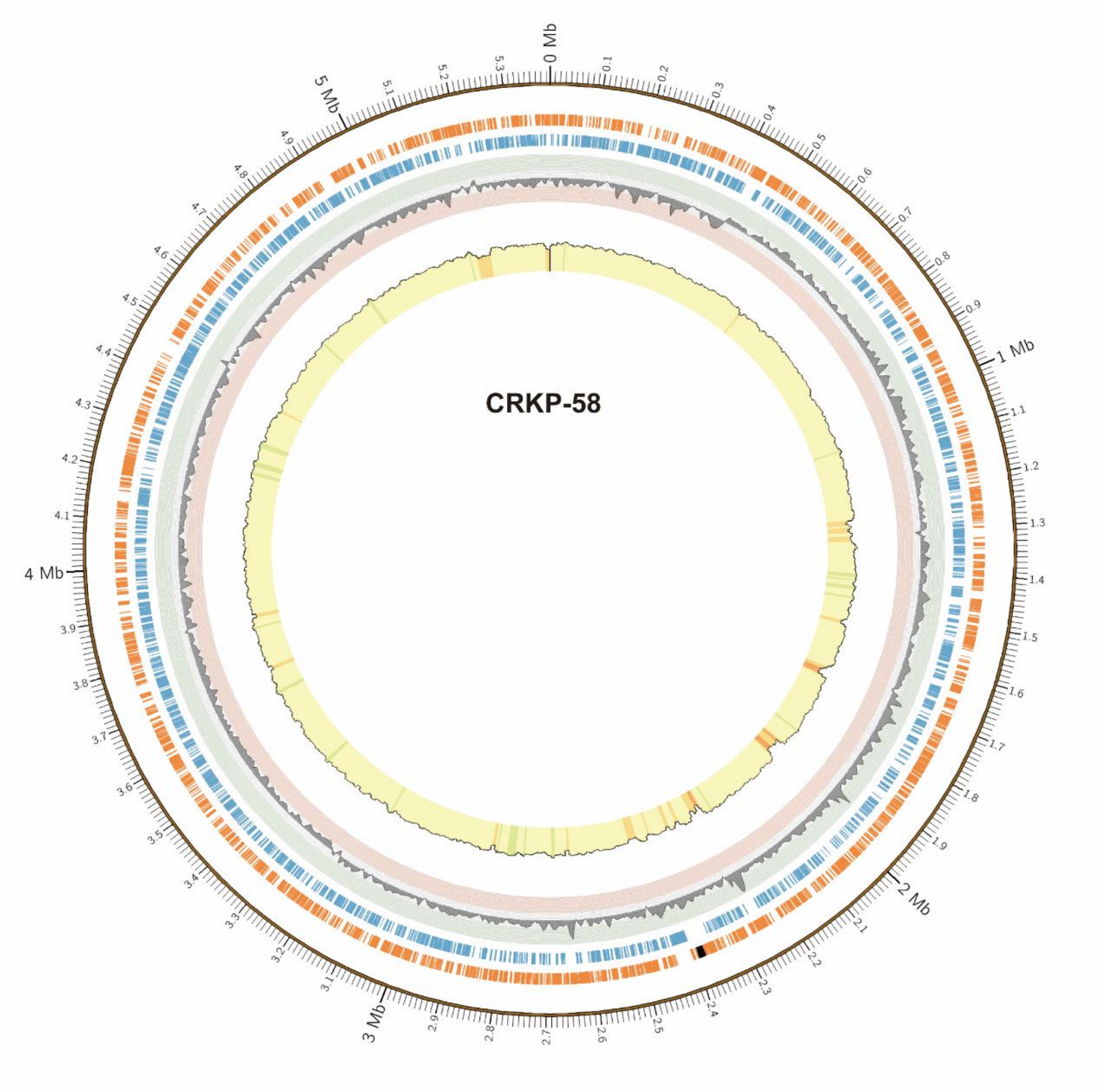

Fig. S8 The circular genome map of CRKP-58 is structured as follows, from the outermost to the innermost layer: First circle: Displays the genome sequence information. Second circle: Shows the CDS, rRNA, and tRNA regions of the reference genome, with the outer layer representing the positive strand and the inner layer the negative strand. Third circle: Presents the GC skew curve of the genome sequence, calculated by a sliding window of 2,000 bp to determine the average GC content, along with a dashed line indicating the GC skew baseline of 0. Fourth circle: Depicts the GC content curve of the genome sequence, also calculated by a sliding window of 2,000 bp to determine the average GC content, with a dashed line showing the average GC content of the reference genome.

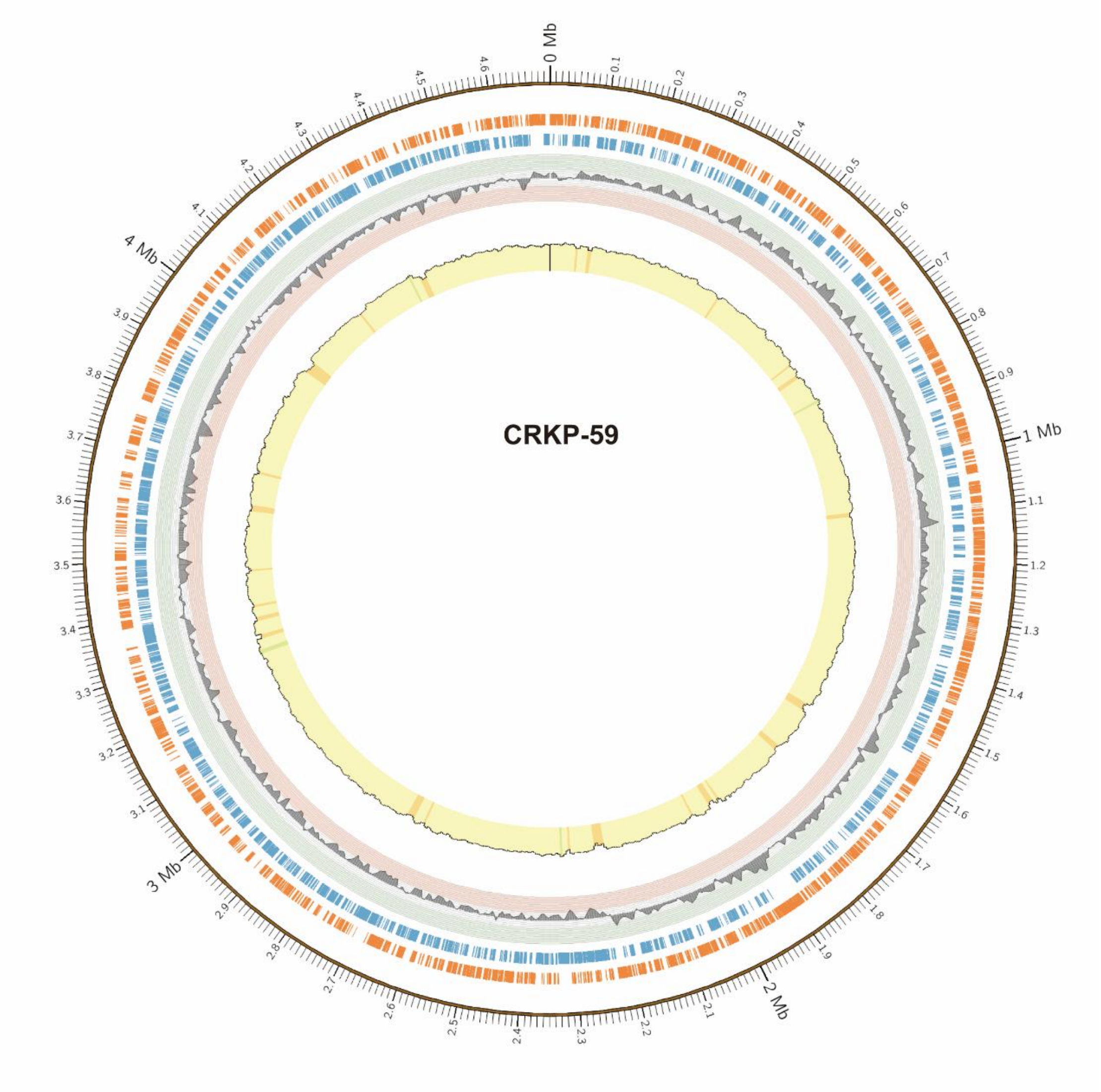

Fig. S9 The circular genome map of CRKP-59 is structured as follows, from the outermost to the innermost layer: First circle: Displays the genome sequence information. Second circle: Shows the CDS, rRNA, and tRNA regions of the reference genome, with the outer layer representing the positive strand and the inner layer the negative strand. Third circle: Presents the GC skew curve of the genome sequence, calculated by a sliding window of 2,000 bp to determine the average GC content, along with a dashed line indicating the GC skew baseline of 0. Fourth circle: Depicts the GC content curve of the genome sequence, also calculated by a sliding window of 2,000 bp to determine the average GC content, with a dashed line showing the average GC content of the reference genome.

Table S2. Sequencing feature.

Strain	Protein coding genes	tRNA genes	Total gene length	Average gene length	GC content	Gene density (genes/Mb)	Gene/ Geonme (%)	Intergenetic region length
CREC-2	4670	95	4467931	933	51%	973	88%	565961
CREC-32	4913	90	4775033	949	58%	956	88%	610091
CREC-36	4785	90	4533536	925	51%	981	88%	574401
CREC-U1	4782	90	4533509	926	51%	981	88%	573989
CREC-U3	4788	90	4534547	925	51%	982	88%	572950
CRKP-1	4786	90	4534253	925	51%	982	88%	573668
CRKP-3	4917	87	4693581	933	58%	972	88%	602700
CRKP-58	5504	90	4723254	840	58%	1068	87%	663295
CRKP-59	4381	87	4235857	942	56%	979	90%	463341

Table S4. Detection of genomic islands in the nine CRE strains.

Strain	GI_ID	SEQ_ID	start	end	GI_length
CREC36	GI_36_1	CRE-36	1160670	1212437	51768
CREC36	GI_36_2	CRE-36	1411091	1490311	79221
CREC36	GI_36_3	CRE-36	1964457	1971287	6831
CREC36	GI_36_4	CRE-36	2572289	2608467	36179
CREC36	GI_36_5	CRE-36	2936870	2941818	4949
CREC36	GI_36_6	CRE-36	3739872	3792823	52952
CREC36	GI_36_7	CRE-36	4166862	4176491	9630
CREC36	GI_36_8	CRE-36	4837787	4929157	91371
CREC2	GI_2_1	CRE-2	998460	1069728	71269
CREC2	GI_2_2	CRE-2	1552090	1578833	26744
CREC2	GI_2_3	CRE-2	3476826	3511481	34656
CREC2	GI_2_4	CRE-2	3865658	3887411	21754
CREC2	GI_2_5	CRE-2	3896190	3951414	55225
CREC2	GI_2_6	CRE-2	4187288	4240978	53691
CREC2	GI_2_7	CRE-2	4263331	4283178	19848
CREC2	GI_2_8	CRE-2	4346936	4350966	4031
CREC2	GI_2_9	CRE-2	4693211	4718643	25433
CREC2	GI_2_10	CRE-2	4724700	4744910	20211
CREC32	GI_32_1	CRE-32	290469	320278	29810
CREC32	GI_32_2	CRE-32	726078	737935	11858
CREC32	GI_32_3	CRE-32	808438	863611	55174
CREC32	GI_32_4	CRE-32	1228945	1266869	37925
CREC32	GI_32_5	CRE-32	1484058	1535015	50958
CREC32	GI_32_6	CRE-32	1861281	1873752	12472
CREC32	GI_32_7	CRE-32	1891659	1955016	63358
CREC32	GI_32_8	CRE-32	1983193	1995883	12691
CREC32	GI_32_9	CRE-32	3079313	3089637	10325
CREC32	GI_32_10	CRE-32	3390861	3398218	7358

CREC32	GI_32_11	CRE-32	3984741	4038663	53923
CREC32	GI_32_12	CRE-32	4065007	4072817	7811
CREC32	GI_32_13	CRE-32	4727205	4766223	39019
CREC32	GI_32_14	CRE-32	4917695	4928429	10735
CRECU1	GI_U1_1	CRE-U1	1582	53509	51928
CRECU1	GI_U1_2	CRE-U1	248965	302414	53450
CRECU1	GI_U1_3	CRE-U1	1641956	1729899	87944
CRECU1	GI_U1_4	CRE-U1	2393473	2401626	8154
CRECU1	GI_U1_5	CRE-U1	2779184	2830908	51725
CRECU1	GI_U1_6	CRE-U1	3628962	3634206	5245
CRECU1	GI_U1_7	CRE-U1	3959673	3999603	39931
CRECU1	GI_U1_8	CRE-U1	5076260	5105711	29452
CRECU3	GI_U3_1	CRE-U3	9324	23057	13734
CRECU3	GI_U3_2	CRE-U3	397096	406725	9630
CRECU3	GI_U3_3	CRE-U3	1068021	1159391	91371
CRECU3	GI_U3_4	CRE-U3	2498842	2550609	51768
CRECU3	GI_U3_5	CRE-U3	2749263	2828483	79221
CRECU3	GI_U3_6	CRE-U3	3302629	3309459	6831
CRECU3	GI_U3_7	CRE-U3	3909584	3946200	36617
CRECU3	GI_U3_8	CRE-U3	4274603	4279551	4949
CRECU3	GI_U3_9	CRE-U3	5077605	5101944	24340
CRKP-3	GI_3_1	CRKP-3	156999	173802	16804
CRKP-3	GI_3_2	CRKP-3	192175	213450	21276
CRKP-3	GI_3_3	CRKP-3	1343579	1351210	7632
CRKP-3	GI_3_4	CRKP-3	1401225	1445414	44190
CRKP-3	GI_3_5	CRKP-3	1981771	1996486	14716
CRKP-3	GI_3_6	CRKP-3	2336470	2356162	19693
CRKP-3	GI_3_7	CRKP-3	3061778	3073468	11691
CRKP-3	GI_3_8	CRKP-3	3825786	3851874	26089
CRKP-3	GI_3_9	CRKP-3	4285471	4301396	15926
CRKP-3	GI_3_10	CRKP-3	4480130	4489512	9383
CRKP-58	GI_58_1	CRKP-58	760953	776963	16011
CRKP-58	GI_58_2	CRKP-58	1184126	1197191	13066
CRKP-58	GI_58_3	CRKP-58	1265680	1321723	56044
CRKP-58	GI_58_4	CRKP-58	1686738	1727147	40410
CRKP-58	GI_58_5	CRKP-58	1945859	1992865	47007
CRKP-58	GI_58_6	CRKP-58	2319325	2331283	11959
CRKP-58	GI_58_7	CRKP-58	2349491	2412848	63358
CRKP-58	GI_58_8	CRKP-58	2441245	2453935	12691
CRKP-58	GI_58_9	CRKP-58	3538777	3545550	6774
CRKP-58	GI_58_10	CRKP-58	3849405	3856762	7358
CRKP-58	GI_58_11	CRKP-58	4522726	4535592	12867
CRKP-58	GI_58_12	CRKP-58	4827015	4857323	30309
CRKP-58	GI_58_13	CRKP-58	5198716	5224198	25483

CRKP-59	GI_59_1	CRKP-59	327512	336292	8781
CRKP-59	GI_59_2	CRKP-59	615538	630547	15010
CRKP-59	GI_59_3	CRKP-59	683120	727776	44657
CRKP-59	GI_59_4	CRKP-59	1712604	1721236	8633
CRKP-59	GI_59_5	CRKP-59	1731155	1739494	8340
CRKP-59	GI_59_6	CRKP-59	2085181	2092387	7207
CRKP-59	GI_59_7	CRKP-59	3348407	3394407	46001
CRKP-59	GI_59_8	CRKP-59	3999042	4030405	31364
CRKP-59	GI_59_9	CRKP-59	4355734	4383188	27455
CRKP-1	GI_1_1	CRKP-1	301995	353762	51768
CRKP-1	GI_1_2	CRKP-1	552416	631633	79218
CRKP-1	GI_1_3	CRKP-1	1105779	1112609	6831
CRKP-1	GI_1_4	CRKP-1	1713173	1749789	36617
CRKP-1	GI_1_5	CRKP-1	2078192	2083140	4949
CRKP-1	GI_1_6	CRKP-1	2881194	2934145	52952
CRKP-1	GI_1_7	CRKP-1	3308184	3317813	9630
CRKP-1	GI_1_8	CRKP-1	3979094	4070466	91373
